# Central Dental Association of Northern New Jersey

**Published:** 1886-05

**Authors:** 


### CENTRAL DENTAL ASSOCIATION OF NORTHERN NEW JERSEY.

Reported for The Independent Practitioner.

A regular meeting was held on Monday evening, March 15, 1886, in the parlors of Dr. William H. Atkinson, 41 East 9th Street, New York, the President, Dr. B. F. Luckey, in the chair.

Dr. Atkinson, not having had time to prepare a paper for the occasion, presented one entitled "Disease," read by him before the Dental Society of the State of New York, in May, 1883. After the reading of the paper he addressed the meeting as follows:

Mr. President, I wish to say a few words in justification of what I have been saying in that paper; and if every one of you could have been with me to-day in the court room, where an effort was made to mulct a dentist in $50,000 damages for inserting what was termed a poisonous substance into a lady's mouth, and thereby producing a myxomatous lupus, that was nominated in various terms by different medical men, you would have been willing to accord me the joy that I have expressed about the growth of dentistry. When I hear men, who said they had taken the degree of doctor of medicine and a pharmaceutical degree, state unblushingly that all combinations of mercury were poisonous and terribly escharotic, that H.G.S., which is the sulphate of mercury, was capable of being dissolved in the mouth and resorbed into the circulation, and of producing salivation, ulceration, alveolar abscess and this specific mode of degeneracy known as lupus or cancer, I began to think they were the boys. It was superficially educated men who swore that this disease was the immediate result of the insertion of an artificial piece, that could not have done anything more than mechanically and thermally disturb the nutrient currents.

Physiologists have said that if you do not know you have got an organ you may be sure you are healthy, but if your attention is called to any organ so that you are reminded that it exists, look out; there is something wrong; the harmonious functional action has been interfered with.

How do they deal with disease? They would make it ease; and they do that by swamping the consciousness. They will give you some narcotic, and make you so drunk that you do not know what is going on. And if they can so confine this stored radiancy in the organism as to make you sweat, or vomit, or purge, then this stored radiancy can come to your relief in its reaction, and they have not only this immediate arresting agency that we call a narcotic, but radical depleting too; and then you come out all right. Is that sound sense or not?

A subcutaneous injection of morphine may be given for any complaint; and it will cure it, too, many times. I had a gravel stone passing from the urethra into the bladder, which put me in terrible agony. I was well posted upon the symptoms of that kind of thing, but I had been accustomed to consider them objectively, and when they became subjective, I didn't know what ailed me. I thought of everything in the neighborhood of the pain, but did not hit the right solution. I sent for my eldest son, who immediately said I had a calculus passing through the urethra, and then I could see he was right. I was trying to think of myself as I had thought of other people, and was taking my feelings in place of my observation. This was a case in which the subcutaneous injection of morphine was legitimate. There was a stone passing through a narrow pipe, and it was giving me intense pain. The morphine was used to produce mental and muscular relaxation by drowning consciousness, and for twenty-four hours I did not know anything at all. The little bit of gravel was passed, and then all I had to do was to feed up. That is a great many years ago, and I have had no trouble of that kind since.

*Dr. Clowes*—Will Dr. Atkinson please tell us what became of the calculus?

*Dr. Atkinson*—It passed through the urethra. There were several little gravels that were broken up. One, I judge, was ragged and sharp, a sort of three cornered calculus, and that stone caused the severest pain. They were less than half the size of a grain of wheat. I have never been conscious of any since. I take on an average, three times a week,one pill of nux vomica and phosphorus and cantharides (McKesson & Robbins' formula) at night. That has a fine effect upon the urinary tract with me.

*Dr. Clowes*—What is its effect?

*Dr. Atkinson*—It is tonic and diuretic if one pill is taken; if I take two it becomes a stimulant, so as to lessen the calibre of the uriniferous tracts.

*Dr. Day*—Did you experience very much nausea?

*Dr. Atkinson*—Not a bit.

*Dr. Day*—If you had, don't you think it would have been some relief?

*Dr. Atkinson*—Yes. That is what I mean by awakening the stored energy in the body. They say the fools all run to a fire. That is just the way it is in the body; all those little mugwumps that have nothing to do, whenever there is an irritation they will run to it. Where the irritation is, there is the flow. The irritation being in a particular territory, there is a call upon this stored radiancy, and when that is impressed upon the stomach it produces nausea by inversion of the peristaltic movement of the intestines and stomach. It is by reason of that inversion that we vomit at all. This would be a benefit, by producing so much relaxation throughout the system that everything would be limp, and then the stone could go on and be expelled.

*Dr. C. S. W. Baldwin*—That juicy pabulum you spoke of in reference to abscessed teeth; is that the same substance that we see after teeth have been extracted, the white substance that is found in their sockets?

*Dr. Atkinson*—Oftentimes it is. It is nothing but broken down tissue that has not been absorbed.

*Dr. Baldwin*—How do you distinguish between healthy and diseased pabulum; between that which is found in the sockets of abscessed teeth and that which is seen after health shows itself, or begins to show itself?

*Dr. Atkinson*—That question is a very hard one to answer, without you go far enough to apprehend what I mean by saying that you cannot have an abscess of any kind in a mammalian body where the blood crassus is normal. An amount of churning must take place in the preparation of the nutrient fluid in the body to admit of its death anywhere, at any point. With patients who are very constipated and jaundiced, with a slow liver, there is unhealthy blood crassus, and then when any little inconvenience occurs, they may have abscesses. If the same kind of obstruction should occur at the foramen of a tooth, that would, in case the blood crassus was bad, produce an abscess and suppuration. If the blood crassus were perfect, what would become of the same obstruction? Simple atrophy would result; a drying up of the contents of the pulp chamber to the end of the root. Then the next thing would be encystment of the end of the root. What constitutes the pulp changes at the foramen? You know there is a little connective tissue that surrounds the leash of blood-vessels and nerves at the end of the root. Are there any absorbents there? I have never found any. A few histologists think they have found absorbents in the pulp. Some think they have found arteries there. I do not think there is an artery in any pulp. What they see are simply blood tracts. Simple atrophy will result in such cases when the pabulum is perfect. Then you will say, who has perfect pabulum? Almost nobody.

*Dr. S. C. G. Watkins*—I would like to ask Dr. Atkinson how we are to determine between health and disease in the case of exposed pulps, and know just when to cap the nerve and be certain that it is living?

*Dr. Atkinson*—The only way that can be known is by testing. The manner of testing is first by the eye. If the pulp be sufficiently exposed, we can very readily see whether there is a drop of pus already formed at the point of exposure. If there is a drop of pus there, the character of that is almost certain to be a favorable indication of saving the pulp. But what would you do if you found pus elsewhere? That is our mistake, that we have thought we were dealing with this point only, when there was really abscess somewhere else in the soft tissues. Remove the pus and then apply almost any of the carbo-hydrates. My preference is the saturated solution of salicylic acid. Then dress it with a little sandarach varnish, pretty well dried down, so as to keep out food and all foreign substances. The next day you will see at once whether the inflammatory action has been arrested. If pus has been formed and you get a drop of pus, it is next to certain that there will be proteinaceous coagulum, and that will make the very best kind of nature's dressing, so as to enable you to cover it up. How would you cover it? A little oxide of zinc mixed into a paste with half creosote and half oil of cloves applied first; then put oxy-phosphate of zinc over that, and instruct the patient to come the next day if there is uneasiness; if perfectly free from pain or uneasiness, the patient need not come for a week. Let it go for some time until satisfied of the extinction of the mischief. If there should be any trouble it is easy to take out the oxy-phosphate filling and re-dress, but never destroy a pulp for that. I know of but one excuse for destroying a pulp, and that is when pulp stones are attached to the sides of the pulp chamber and you cannot take them out. The first pulp I have destroyed in the city of New York has occurred within three weeks, and that was my excuse for doing it. It was in a left superior third molar. I did put in arsenic, and extirpated the body of the pulp with the pulp stone.

*Dr. Watkins*—Where the pulp is a little exposed and there is considerable inflammation, but no drop of pus, what would you do?

*Dr. Atkinson*—Bleed it.

*Dr. Watkins*—How can we be morally certain of saving that pulp alive by capping?

*Dr. Atkinson*—I am only morally certain of going down stairs legitimately, by not attempting to go down at one step. Take the steps in their proper order. The first step to take is to relieve the congestion. What is the best method of relieving congestion in a pulp? It is to excise the exposed part and let it bleed freely. Don't kill the pulp for the sake of having a funeral, but for humanity's sake, give the little fellow a chance to live. If it dies, then bury it, or cremate it.

*Dr. Watkins*—What percentage of exposed pulps do you save?

*Dr. Atkinson*—When I was keeping percentages, it was so high that almost everybody said I exaggerated. I do not think one per cent. failed. I think a pulp that is congested is capable of being preserved. My reason is this; whenever congestion is carried to the point of transudation of pabulum or of blood, there is a more or less arrest of the circulation that needs relief, and when that is responded to, and the other portions are sufficiently fluid to keep up their wonted circulation around these little ponds or lakes—they are like varicose veins—then cut that out, and it recuperates itself by simple treatment. You will be astonished to see how many pulps will be preserved, even where you have taken away the entire body of the pulp, leaving only that which is in the canals of the roots, especially the palatal roots of the upper teeth.

*Dr. Meeker*—I want to ask Dr. Atkinson about the, to him, familiar subject of sponge-grafting. At our last meeting I spoke of a case that I had just started that day, or the day previous. During the interval I have had three pieces of sponge in the cavity, and none of them have stayed. It was a case of two lower incisors. I burred away the necrosed portions, and cut off the apexes of the roots. New tissue has formed there, and it is nearly healed over, but the sponge-grafts that I inserted would not stay.

*Dr. Atkinson*—What kind of a capping did you have?

*Dr. Meeker*—A little gutta-percha.

*Dr. Atkinson*—That is the trouble. You must have a pocket that is equivalent to the cavity in the flesh, where the tissue has been removed, before you can expect that exudant that forms the clot out of which new tissue comes shall penetrate the entire meshes of the sponge. I have a case that I just clipped yesterday, a left superior incisor, that has not had a drop of pus since a week ago yesterday. I saw that the pabulum did not quite fill the sponge, and I clipped off that part to where the granulation came up and boiled out. Take an impression and fit a plate of thin platinum, or Reese base, something that will make a pocket tight enough to keep out food. Have the sponge fitted so as not to press too closely upon the walls of the cavity and thereby shut out the exudant, but so arranged as to hold the exuding pabulum.

*Dr. Meeker*—The new tissue is all formed—I can see it—and without the sponge, and I do not understand it.

*Dr. Atkinson*—Your pocket has not been properly protected; your sponge was operating like a scab. When you pulled the sponge out, did blood follow?

*Dr. Meeker*—Yes. It is growing smaller every day.

*Dr. Atkinson*—Did not some part of the sponge remain in there, being converted into tissue?

*Dr. Meeker*—That was my impression. It did not seem as though I took it all out.

*Dr. Atkinson*—It seems to be converted into tissue. In almost all my early cases, especially when I used as high as 180° or 200° F. in sterilizing, the sponge would crumble away, but since I do not carry it above 160°, I have not had any difficulty of that kind. As sure as I get fresh pabulum so as to fill into the sponge, I have no trouble; the sponge serves as the scaffold poles upon which to build new tissue.

*Dr. M. L. Rhein*—I understand the spong is sterilized at 180° or 160°. Why is that degree of heat necessary?

*Dr. Atkinson*—In incubation, the incubating range is from 95 to 105 or 110 degrees; when you come to cook albumen, you will find it will begin to fibrilize at 120°. It will be very tender and will break under the dissecting needle until you get it up to 163° or 164°, and then you can begin to lift it. When it will lift it is no longer fit for the reproduction of embryonal tissue. It is my belief that the coagulation of the albuminous part of the sponge at that high degree of heat unfits it for conversion into tissue.

*Dr. Rhein*—In bringing the heat up to 160°, is there not a great risk of carrying it higher?

*Dr. Atkinson*—There is no trouble at all. Have a Bunsen burner, and put your instrument in there and watch it.

*Dr. Rhein*—Would it be well to carry it above 105°? What advantage is there in carrying it above that point?

*Dr. Atkinson*—I believe there are certain microbes that are not destroyed until the heat is raised to about 130°.

*Dr. Rhein*—I made failures in some of my sponge grafts, and the only reason I could assign was that the sponge was sterilized at too high a temperature.

*Dr. Atkinson*—How much heat did you employ?

*Dr. Rhein*—I only estimated it, and did not use a thermometer. I did not bring the water to the boiling point. How do you know exactly the temperature at which you sterilize?

*Dr. Atkinson*—I have a small annealed glass that will stand heat; I put the sterilizing solution into that and place the sponge in it; also a thermometer and set the glass into boiling water or over a Bunsen burner. I watch it until it gets up to 130°, then leave it until it goes up 5 or 6 degrees more. I have been showing so many how to do it that I have become *au fait* in the matter. You must be sure that the thermometer is properly registered.

*Dr. Rhein*—I have met with that difficulty. Lately I have been using warm water to sterilize the sponge, and I have met with perfect success. I do not carry the heat much over one hundred deg.

*Dr. Atkinson*—I do not know why you would not put a clean sponge in without cooking it at all.

*Dr. Rhein*—I do not see the advantage of bringing the heat higher. In one case I thought I was getting a beautiful result for about ten days, and then instead of getting pabulum I had ichor, just the opposite; disintegration of the sponge instead of forming a support, and I had to commence at the beginning again.

